# Morphological Characteristics of Motor Neurons Do Not Determine Their Relative Susceptibility to Degeneration in a Mouse Model of Severe Spinal Muscular Atrophy

**DOI:** 10.1371/journal.pone.0052605

**Published:** 2012-12-20

**Authors:** Sophie R. Thomson, Joya E. Nahon, Chantal A. Mutsaers, Derek Thomson, Gillian Hamilton, Simon H. Parson, Thomas H. Gillingwater

**Affiliations:** 1 Euan MacDonald Centre for Motor Neurone Disease Research, University of Edinburgh, Edinburgh, United Kingdom; 2 Centre for Integrative Physiology, University of Edinburgh, Edinburgh, United Kingdom; National Institute of Health, United States of America

## Abstract

Spinal muscular atrophy (SMA) is a leading genetic cause of infant mortality, resulting primarily from the degeneration and loss of lower motor neurons. Studies using mouse models of SMA have revealed widespread heterogeneity in the susceptibility of individual motor neurons to neurodegeneration, but the underlying reasons remain unclear. Data from related motor neuron diseases, such as amyotrophic lateral sclerosis (ALS), suggest that morphological properties of motor neurons may regulate susceptibility: in ALS larger motor units innervating fast-twitch muscles degenerate first. We therefore set out to determine whether intrinsic morphological characteristics of motor neurons influenced their relative vulnerability to SMA. Motor neuron vulnerability was mapped across 10 muscle groups in SMA mice. Neither the position of the muscle in the body, nor the fibre type of the muscle innervated, influenced susceptibility. Morphological properties of vulnerable and disease-resistant motor neurons were then determined from single motor units reconstructed in Thy.1-YFP-H mice. None of the parameters we investigated in healthy young adult mice – including motor unit size, motor unit arbor length, branching patterns, motor endplate size, developmental pruning and numbers of terminal Schwann cells at neuromuscular junctions - correlated with vulnerability. We conclude that morphological characteristics of motor neurons are not a major determinant of disease-susceptibility in SMA, in stark contrast to related forms of motor neuron disease such as ALS. This suggests that subtle molecular differences between motor neurons, or extrinsic factors arising from other cell types, are more likely to determine relative susceptibility in SMA.

## Introduction

Spinal muscular atrophy (SMA) is the most common childhood form of motor neuron disease affecting 1∶6,000–1∶10,000 live births [1]. SMA causes the degeneration of lower motor neurons leading to muscle atrophy, progressive paralysis and eventually premature death [2], [3]. SMA is clinically divided into four main sub-types (type I, II, III and IV), based on the age of onset and severity of symptoms [2]. Type I is the most severe form, with patients exhibiting disease symptoms before six months of age and death occurring in the first two years of life [2].

SMA is caused by reduced levels of the ubiquitously expressed Survival Motor Neuron (SMN) protein [4]. This results from the deletion or mutation of the Survival Motor Neuron 1 gene (*SMN1*) with retention of a near identical gene (*SMN2*) only capable of producing low levels of full-length SMN protein [5]. Therefore, the copy number of *SMN2* determines disease severity, with higher copy numbers resulting in increased levels of full length SMN protein and less severe disease phenotypes [6].

Previous studies have shown that one of the earliest pathological events affecting motor neurons in SMA is a breakdown of motor nerve terminals at the neuromuscular junction (NMJ), rendering skeletal muscle fibres denervated [7]–[11]. Interestingly, the rate at which NMJs degenerate in mouse models of severe SMA varies considerably between different muscles, indicating differing levels of vulnerability between distinct pools of motor neurons. For example, we have previously reported that even within a single anatomically-defined muscle, the levator auris longus (LAL), motor neurons innervating the two distinct muscle bands were affected differently, with those innervating the rostral band (LALr) being unaffected, while those innervating the caudal band (LALc) were severely affected [10]. Similarly, a study by Ling and colleagues revealed a broad spectrum of NMJ vulnerability between distinct pools of motor neurons in the Δ7 mouse model of SMA [12]. It is not yet clear what determines whether the motor neurons innervating a particular muscle are resistant or vulnerable to degeneration in SMA. Findings from recent studies suggested that motor neurons with a delayed-synapsing developmental phenotype may be associated with resistance to the disease, but this appears to be more of a modifying factor (when all other factors are constant) rather than a major determinant of vulnerability [10], [12].

We set out to establish whether core intrinsic morphological features of motor neurons, such as motor unit size or branching patterns, predisposed them to degeneration in SMA. We chose to investigate motor neuron morphology as a possible regulator of vulnerability in SMA as a result of recent evidence obtained during studies of the adult onset form of motor neuron disease, amyotrophic lateral sclerosis (ALS). A growing body of evidence suggests that SMA and ALS share biochemical pathways [13]–[15], indicating that factors regulating relative motor neuron susceptibility in ALS may also underlie susceptibility in SMA. In ALS there is a growing body of evidence suggesting that large motor units are the first to degenerate [16]–[20]. For example, electromyographical (EMG) data from ALS patients showed that the largest and strongest motor units were preferentially affected [17]. This evidence has been replicated in animal models of ALS, such as the SOD1^G93A^ mouse, where large diameter motor axons were preferentially decreased in number in the ventral roots [18]. EMG data has similarly shown that larger motor units innervating fast-twitch muscles degenerate during the earliest stages of the disease [19]. Moreover, there is also evidence that the large motor neurons that are preferentially affected in ALS are also more vulnerable to age-related changes in healthy mice [20].

Here, we report that, in sharp contrast to ALS, morphological characteristics of motor neurons, such as motor unit size, do not determine their relative vulnerability to degeneration in SMA.

## Materials and Methods

### Ethics Statement

All animal experiments were approved by a University of Edinburgh internal ethics committee and were performed under license by the UK Home Office (project license number 60/3891).

### Mice

*Smn+/−;SMN2* mice (Jackson labs strain no. 005024) on a congenic FVB background were maintained as heterozygote breeding pairs under standard SPF conditions in animal care facilities in Edinburgh. All animal procedures and breeding were performed in accordance with Home Office and institutional guidelines. Litters produced from SMA colonies were retrospectively genotyped using standard PCR protocols (JAX® Mice Resources).

Thy.1-YFP-H mice [21] on a congenic C57Bl/6 background were originally obtained from Jackson Laboratories and were maintained under standard SPF conditions in animal care facilities in Edinburgh.

### Muscle Preparation

Neonatal *Smn−/−;SMN2* [22] (P5) and unaffected littermates (P5, P7 and P14) were killed by intra-peritoneal injection of sodium pentabarbitol. Required muscles were dissected in 0.1 M phosphate buffered saline (PBS). Whole mount muscles (LAL, AAL, AS, IS, TS, TVA) were fixed in 0.1 M PBS containing 4% paraformaldehyde (Electron Microscopy Studies) for 10 min at room temperature. Muscles were then processed for immunohistochemical staining (see below). For hind limb muscles, the skin was removed and the limbs were fixed for 15 minutes in 0.1 M PBS containing 4% paraformaldehyde at room temperature. After fixation, the muscles were dissected from the limb. Hind limb muscles were then cryo-protected in 0.1 M PBS containing 30% sucrose overnight at 4°C and sectioned at 100 µm on a freezing microtome after embedding in OCT. Hind limb muscles were then stored in cold 0.1 M PBS and stained using the same immunohistochemistry protocol as the whole mount muscles.

Thy.1-YFP-H [21] mice of approximately 22 weeks of age were killed by overdose of anaesthetic via inhalation. Only whole mount muscle preparations were used for tracing motor units and the TVA was excluded from analysis due to poor YFP expression in all muscles examined. The muscles were dissected in 0.1 M PBS then exposed to TRITC-conjugated α-bungarotoxin (5 mg/ml) for 10 minutes and then fixed in 0.1 M PBS containing 4% paraformaldehyde for 15 minutes at room temperature. The muscles were then mounted in Mowiol (Calbiochem) on glass slides and coverslipped for subsequent imaging.

### Immunohistochemistry

Muscles were permeabilised in 2% Triton X in 0.1 M PBS for 30 minutes and then blocked in 4% bovine serum albumin and 1% Triton X in 0.1 M PBS for 30 minutes. For endplate occupancy and synapse elimination counts, muscles were then incubated overnight at 4°C in primary antibodies against neurofilament (anti-mouse 2H3) (1∶200 dilution; Developmental Studies Hybridoma Bank) and anti-mouse SV2 (1∶100 dilution; Developmental Studies Hybridoma Bank) diluted in blocking solution. For Schwann cell counts, muscles were incubated overnight at 4°C in primary antibody anti-rabbit S100 (1∶400 dilution; Dako). After washing for 2 h with four changes of 0.1 M PBS, muscles were incubated with 5 mg/ml TRITC-conjugated α-bungarotoxin (Biotium, Inc) for 10 minutes at room temperature. Muscles were then washed in 0.1 M PBS for 5 minutes. Muscles for NMJ counts and synapse elimination studies were then incubated with 1∶100 dilution of Donkey anti-Mouse Dylight488 (H+L) (Stratech Scientific) in 0.1 M PBS for 4 h at room temperature. Muscles for Schwann cell counts were incubated with a 1∶60 dilution of Swine anti-rabbit FITC (Dako) in 0.1 M PBS for 4 hours at room temperature. Muscles were then washed for 30 minutes in three changes of 0.1 M PBS. Muscles for Schwann cell counts were then further incubated with TO-PRO3 for 10 minutes and washed with 0.1 M PBS. Muscles were then mounted in Mowiol (Calbiochem) on glass slides and coverslipped for subsequent imaging.

### Imaging and Analysis

Muscle preparations were viewed using a standard epifluorescence microscope equipped with a chilled CCD camera (10×objective; 0.3NA; Nikon IX71 microscope; Hammamatsu C4742-95, OpenLab software), and a laser scanning confocal microscope (20×objective/0.4NA; 40×objective/1.3NA oil objective; 63×objective/1.4NA oil objective; Zeiss LSM 710 confocal). TRITC labelled preparations were imaged using 543 nm, excitation and 590 nm emission optics; YFP-labelled preparations were imaged using 488 nm excitation and 520 nm emission optics. For confocal microscopy, 488 nm, 543 nm and 633 nm laser lines were used for excitation and confocal Z-series were merged using Zen software.

### Reconstruction of Motor Units

Reconstructions of whole motor units were obtained by montaging fluorescent micrographs from TRITC-conjugated α-bungarotoxin and YFP-H labelled muscle preparations in Adobe Photoshop software. Individual motor neurons were traced by hand and arbor lengths and endplate areas were measured using ImageJ software.

### Quantification and Analysis

For synaptic vulnerability studies, a minimum of 80 endplates per muscle per mouse, selected at random, were assessed in each muscle preparation. Muscles with poor staining and/or damage were excluded from further analysis. For occupancy counts, the occupancy of individual NMJs was evaluated by categorising endplates as either fully occupied (neurofilament and SV2 entirely overlie the endplate), partially occupied (neurofilament and SV2 cover less than 50% of the endplate), or vacant (no neurofilament or SV2 overlying the endplate).

Motor unit reconstructions were used to quantify morphological parameters of single motor neurons. Motor unit size was determined by identifying the number of motor end plates per axon from traced reconstructions. The total intramuscular arbor length of motor units was determined by tracing the axons in the reconstructed images by hand from the point of entering the muscle all the way to the nerve terminals. The arbor length of the axons was determined as the sum of the length of the primary axon trunk and all the distal branches.

The number of branch points in individual motor units was evaluated by marking the branch points by hand in the motor unit reconstructions using Adobe Photoshop. Where more than one motor unit was labelled with YFP, differences in cytosolic levels of YFP expression were used to distinguish between individual motor units. The branching pattern of the axons was further investigated by recreating schematic branching diagrams based on the whole motor unit reconstructions. The branch order for each terminal branch was determined by counting the number of branch points between the nerve terminus and the site where the axon entered the muscle.

End plate area was determined from the Maximum Intensity Z-stack images. The outline of the motor endplates was manually traced in the Image J software to enable the software to calculate the area.

To evaluate the number of axonal inputs in neonatal muscles, the number of axons converging on a single endplate was counted. A minimum of 80 endplates per muscle were counted from each mouse.

The number of terminal Schwann cells per motor end plate was quantified by analysing Maximum Intensity confocal projection images in Adobe Photoshop. To be counted as a tSC, the cell had to be positive for S100 with a TO-PRO-3 labelled nucleus.

### Statistical Analysis

All data were collected into Microsoft Excel spreadsheets and analysed using GraphPad Prism software. Figures were produced on Adobe Photoshop. All bar charts shown are mean ± SEM. Statistical significance was considered to be p<0.05 for all analyses. Individual statistical tests used are detailed in figure legends.

## Results

### Characterisation of Motor Neuron Vulnerability Across a Range of Skeletal Muscles in a Mouse Model of Severe SMA

**Figure 1:**
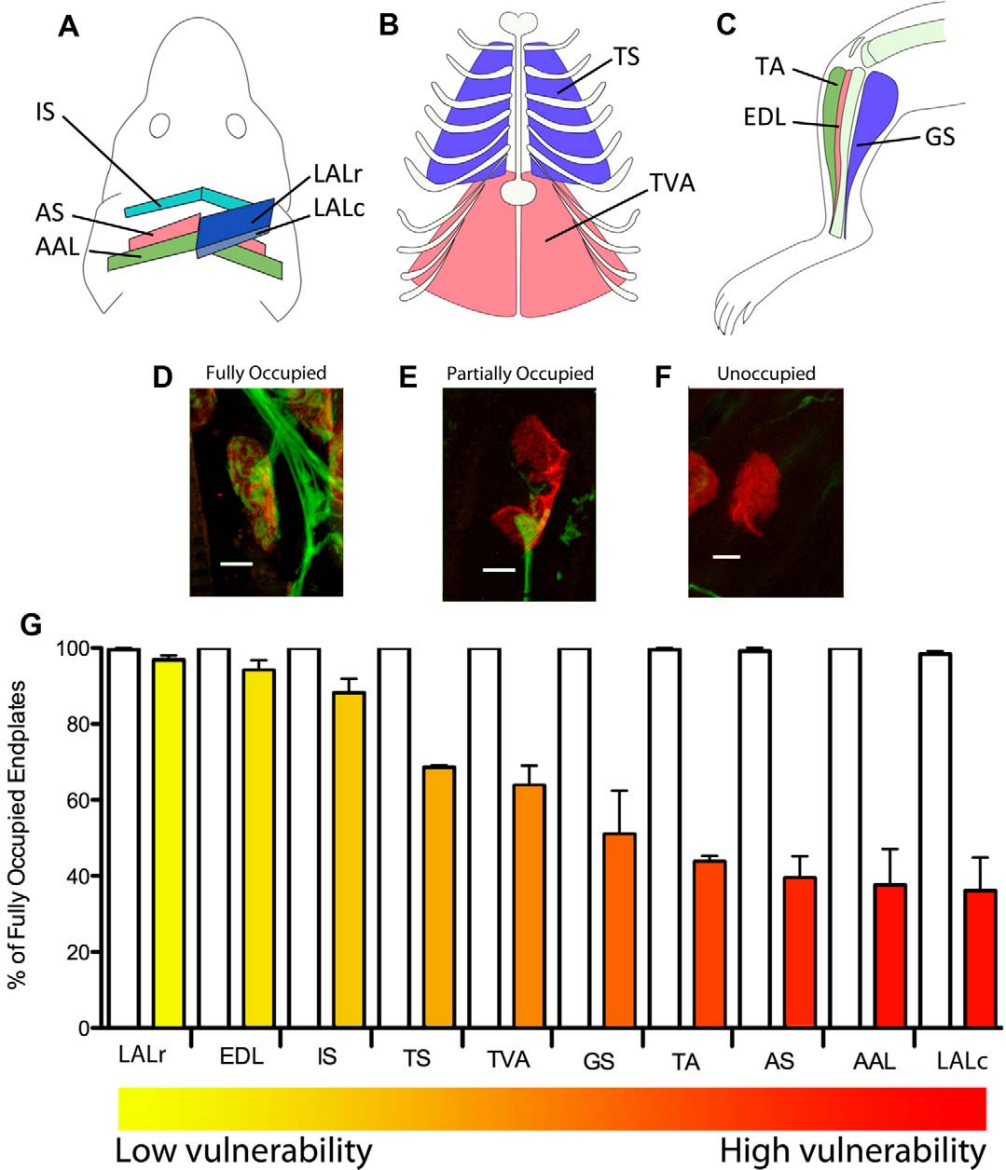
Differential susceptibility to degeneration between motor neurons innervating anatomically distinct muscles in a mouse model of severe SMA. A – Schematic illustration of the anatomical locations of the LALr, LALc, AAL, AS, and IS muscles in the mouse, collectively known as the cranial muscles (Figure adapted from Murray et al., 2010). B – Schematic illustration of the anatomical locations of the TVA and TS muscles in the abdominal and thoracic walls of the mouse. C – Schematic illustration of the anatomical locations of the TA, EDL and GS muscles in the hind limb of the mouse. D−F – Representative confocal micrographs showing differing levels of synaptic pathology at neuromuscular junctions in P5 *Smn−/−;SMN2* mice (green = neurofilament and SV2; red = bungarotoxin-labelled acetylcholine receptors). D shows an example of a healthy, fully occupied motor endplate. E shows an example of a partially occupied motor endplate, where the motor nerve terminal (green) has retracted from the majority of the motor endplate. F shows an example of a vacant motor endplate where the nerve terminal has completely retracted from the motor endplate. Scale bars = 5 µm. G – Bar chart (mean±SEM) showing the percentage of fully occupied endplates in healthy littermate controls (white bars; N = 3 mice) and *Smn−/−;SMN2* mice (coloured bars; N = 3 mice). Mean values were used to rank the muscles from low vulnerability (yellow) to high vulnerability (red). This colour coding system has been applied to subsequent figures in order to distinguish muscles with vulnerable and disease-resistant motor neurons.

In order to determine the extent of heterogeneity in motor neuron pathology occurring between distinct pools of motor neurons in a severe mouse model of SMA (*Smn−/−;SMN2*; [22]), we initially quantified NMJ pathology in ten anatomically distinct skeletal muscles from various body regions at a late-symptomatic time-point (postnatal day 5; P5). We selected muscles from the cranial region, torso and hind limb: levator auris longus (LAL) (subdivided into caudal (LALc) and rostral (LALr) bands), abductor auris longus (AAL), auricularis superior (AS) and interscutularis (IS) (Figure 1A; see [23]); triangularis sterni (TS) and transversus abdominis (TVA) (Figure 1B); tibilais anterior (TA), extensor digitorum longus (EDL) and gastrocnemius (GS) (Figure 1C). As expected, all of the muscles examined showed evidence of fully occupied (Figure 1D), partially occupied (Figure 1E) and vacant endplates (Figure 1F), albeit in varying proportions (Figure 1G). The percentage of fully occupied (i.e. healthy) endplates was then used to rank muscles with respect to the severity of NMJ pathology/motor neuron degeneration (Figure 1G). We used a ‘heat-map’ approach, where red indicates the most severely affected motor neurons, to colour-code individual muscles according to the relative vulnerability of their innervating motor neurons (Figure 1G). The colour assigned to each muscle was retained for use in further correlation analyses.

In agreement with previous studies on mouse models of severe SMA [10]–[12], neither the position of the muscle in the body (e.g. axial versus appendicular), nerve stump length (e.g. distance from the spinal cord), nor muscle fibre type correlated with the severity of motor neuron pathology. For example, both the LALc and LALr are composed almost exclusively of fast twitch muscle fibres and are located the same distance from the CNS ([24]; Figure 1G; Table 1), but were at opposite ends of our vulnerability spectrum. A similar scenario was observed for the EDL and TA muscles ([25] (Figure 1G; Table 1).

**Table 1:** Muscle fibre twitch types of muscles analysed from *Smn−/−;SMN2* mice.

Muscle	Twitch Type
LALr	Fast
EDL	Fast
IS	–
TS	Slow
TVA	Slow
GS	Mixed
TA	Fast
AS	Slow
AAL	Fast
LALc	Fast

Table 1. Table showing the muscle fibre type for muscles collected from *Smn−/−;SMN2* mice (see Figure 1G). Muscles are ranked in order of vulnerability to SMA, from low at the top to high at the bottom. Muscle fibre type is based on data from previously published studies [10], [23], [25].

### Intrinsic Morphological Characteristics of Motor Neurons do not Correlate with Susceptibility to Disease

In order to address whether intrinsic morphological features of vulnerable and disease-resistant motor neurons correlated with their relative susceptibility in SMA, we reconstructed entire single motor units innervating a range of vulnerable and disease-resistant muscles in healthy young adult Thy.1-YFP-H [21] mice. YFP-H mice express yellow fluorescent protein (YFP) in a small subset of motor neurons [21], making it possible to identify, trace and reconstruct single motor units in the confocal microscope. We chose to perform these experiments in healthy mice, rather than SMA mice, to ensure that we were comparing intrinsic morphological characteristics of motor neurons, rather than their responses to pathological stimuli in SMA.

**Figure 2:**
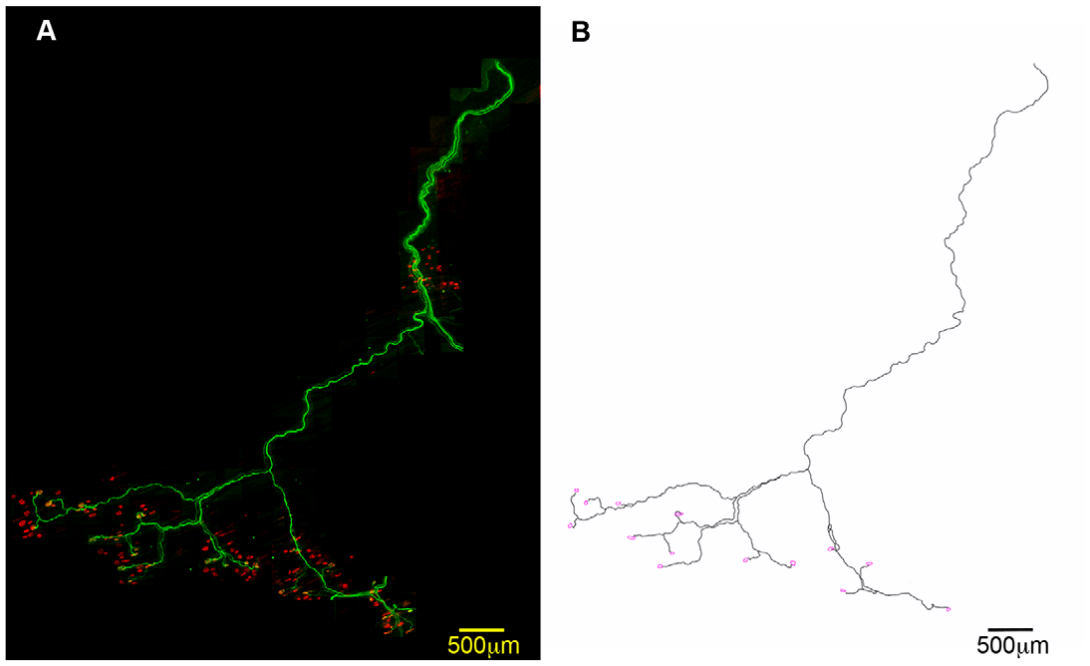
Reconstruction of entire single motor units from Thy.1-YFP-H mice. A – Representative example of a low magnification confocal montage showing YFP-expressing motor neurons innervating the LALr muscle from a Thy.1-YFP-H mouse. Whole mount muscles were dissected and incubated with TRITC-conjugated α-bungarotoxin to label motor endplates (red). B – An example trace of one motor unit from the LALr shown in panel A. A total of 105 entire motor unit reconstructions were produced for subsequent analyses of motor unit morphology.

To assess and quantify morphological characteristics of individual motor neurons, whole motor unit reconstructions were created by montaging fluorescent micrographs of 105 individual motor units (Figure 2A; N≥3 and n≥3 for each muscle; see methods). The reconstructed motor units were subsequently traced using ImageJ software to enable further quantitative analysis (Figure 2B). Only muscles where we could whole-mount the entire preparation were used for these experiments. Attempts to reconstruct whole motor units from muscles that required sectioning before imaging (e.g. gastrocnemius) were unsuccessful, so these muscles were not used for further experiments.

**Figure 3:**
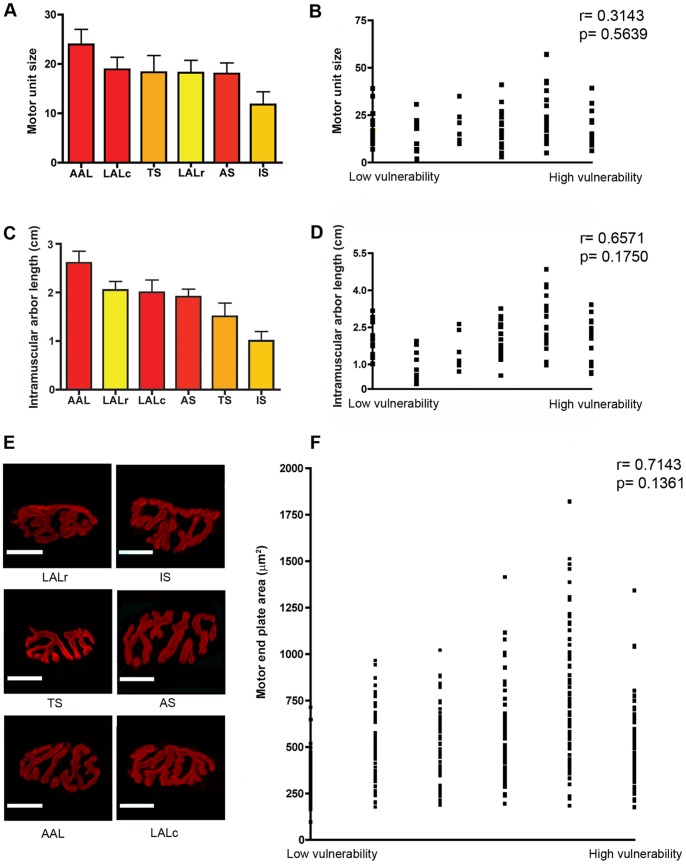
No apparent correlation between morphological properties of motor neurons and their susceptibility to SMA. A/B – Bar chart (Mean±SEM; A) and scatter plot (B) of motor unit sizes in a range of vulnerable (red bars in A) and disease-resistant (yellow bars in A) muscles, determined by obtaining the total number of synapses formed by a single motor neuron. No significant correlation was found between motor unit size and the relative susceptibility of a motor neuron (P>0.05, Spearman correlation analysis; N≥3 mice per muscle). C/D – Bar chart (C) and scatter plot (D) of total intramuscular arbor lengths from motor neurons innervating a range of vulnerable and disease-resistant muscles. No significant correlation was found between total intramuscular arbor length and the relative susceptibility of a motor neuron (P>0.05, Spearman correlation analysis; N≥3 mice per muscle). E – Example fluorescence micrographs of motor endplates from each muscle group investigated. Scale bars = 30 µm. F – Scatter plot of endplate areas (µm^2^) in a range of vulnerable and disease-resistant muscles. No significant correlation was found between the size of neuromuscular synapses and the relative susceptibility of innervating motor neurons (P>0.05, Spearman correlation analysis; N≥3 mice per muscle).

First, we wanted to determine whether motor unit size correlated with SMA pathology, thereby testing whether larger motor units were more vulnerable to SMA pathology (c.f. ALS; [16]–[20]). Therefore, we quantified the total number of synapses formed by individual motor neurons innervating each muscle. Whilst there was considerable variability in motor unit size between the different muscles examined (Figure 3A), no significant correlation was found between motor unit size and the relative vulnerability of a motor neuron to SMA (Figure 3A–B). For example, muscles with a high level of vulnerability to SMA were found with both relatively large (AAL) and relatively small (AS) motor units. Similarly, muscles with a low vulnerability could be identified with relatively large (TS) and relatively small (IS) motor unit sizes.

Second, we sought to determine whether there was any correlation between the total length of a motor neuron’s intramuscular axon arbor and its relative vulnerability or resistance to SMA pathology. To this end, all intramuscular axons belonging to a single motor unit were manually traced in ImageJ to enable the measurement of total arbor length. We measured the length of the axon from the point of muscle entry down to the tip of all terminal branches at individual neuromuscular junctions. There was no significant correlation between the total intramuscular arbor length and the susceptibility of a motor neuron to SMA (Figure 3C–D).

Third, we wanted to determine whether there was any correlation between the size or form of neuromuscular synapses formed by individual motor neurons and their susceptibility to SMA. Morphological parameters of individual neuromuscular junctions formed by a motor neuron were assessed by qualitative and quantitative assessment of post-synaptic acetylcholine receptors in reconstructed single motor units. There were no overt qualitative differences between the gross morphology of motor endplates in vulnerable and disease-resistant muscles (Figure 3E). Similarly, we did not observe a correlation between endplate area and vulnerability to synaptic pathology in SMA (Figure 3F).

**Figure 4:**
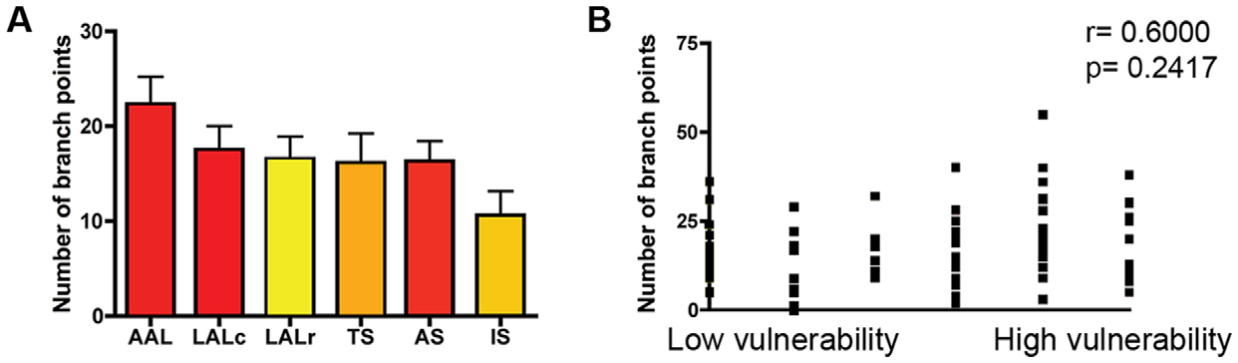
Motor unit branching patterns do not influence susceptibility to degeneration in SMA. A/B – Bar chart (mean±SEM; A) and scatter plot (B) showing the number of branch points in motor units from a range of vulnerable (red bars in A) and disease-resistant (yellow bars in A) muscles. No significant correlation was found between the number of branch points and the relative susceptibility of a motor neuron (P>0.05, Spearman correlation analysis; N≥3 mice per muscle).

**Figure 5:**
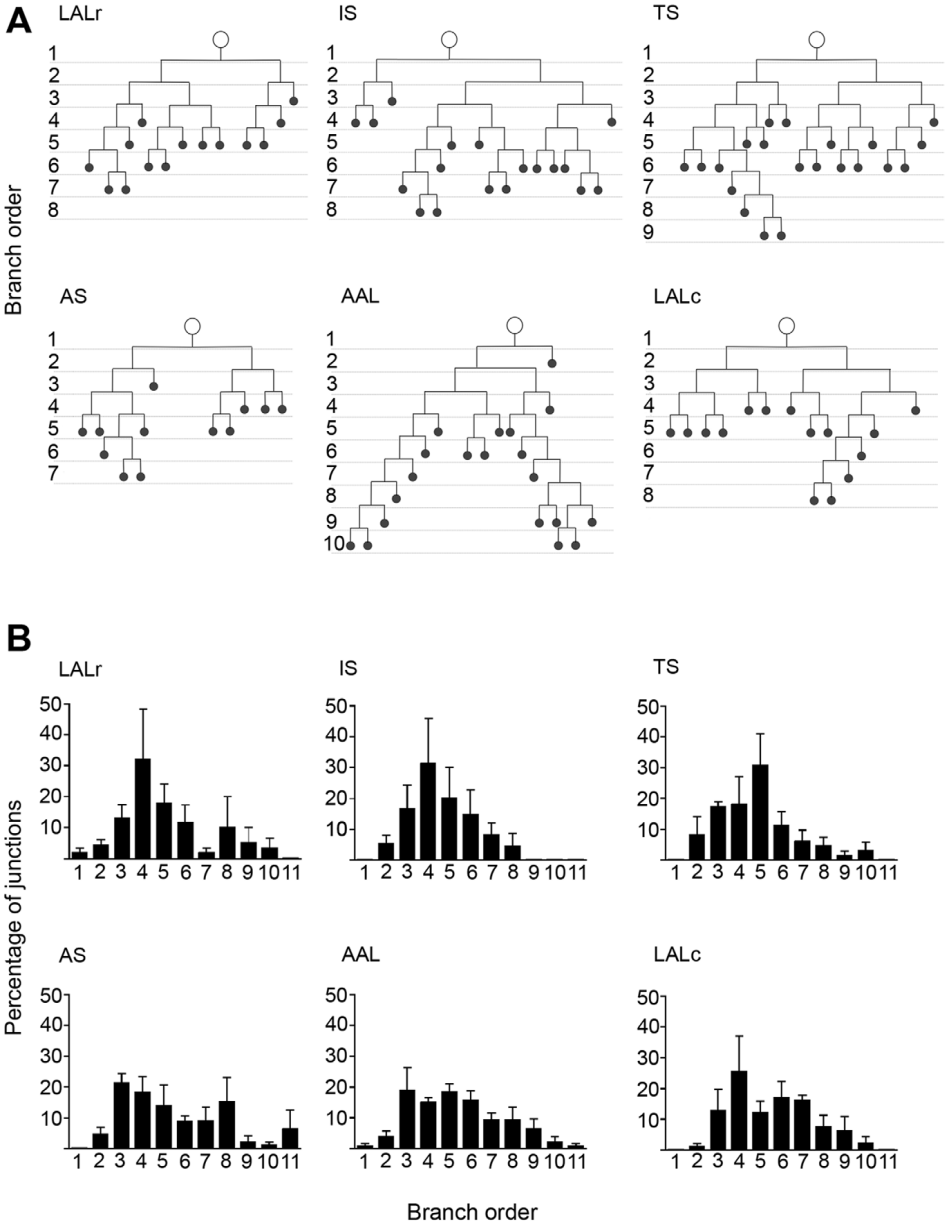
Further analysis of motor unit branching patterns revealed no influence on the susceptibility to degeneration in SMA . A – Representative examples of individual branching diagrams from single motor units innervating the range of vulnerable and disease-resistant muscles analysed. Note the similarities in overall branching patterns in all examples shown. B – Bar charts (mean±SEM) showing the percentage of branch points per branching order in motor units innervating the range of vulnerable and disease-resistant muscles analysed. Once again, note the similarity in distribution of branch orders in all examples shown.

Fourth, we asked whether the branching pattern of individual motor neurons had an impact on their relative susceptibility to SMA. By examining branching patterns of motor neurons in ALS, Valdez and colleagues showed that the distribution of NMJs, with respect to their branch points within the motor unit, influenced their vulnerability [20]. Therefore, to analyse motor neuron branching patterns in motor units with differing susceptibilities to SMA we initially quantified the number of branch points within individual motor units. There was no significant correlation between the number of branch points and the susceptibility of a motor neuron to SMA pathology (Figure 4A–B). We subsequently constructed branching diagrams for individual motor units from a range of vulnerable and disease-resistant muscles. Representative reconstructions of single motor units from each of the six muscles examined are shown in Figure 5A. Comparison of the percentage of branch points per branching order revealed no difference between vulnerable and disease-resistant motor neurons (Figure 5B).

Taken together, these findings suggest that core intrinsic morphological properties of motor neurons are not a key factor determining a motor neuron’s relative susceptibility to degeneration in SMA.

### Motor Neuron Susceptibility in SMA is not Determined by Intrinsic Differences in Synaptic Plasticity

At birth, the majority of neuromuscular junctions receive inputs from more than one axonal input. Over the three weeks after birth, these supernumerary inputs asynchronously withdraw from motor endplates via a dynamic process known as synapse elimination, thereby establishing the mono-innervation pattern characteristic of mature neuromuscular junctions [26]. Given that there are considerable similarities between the processes of synaptic degeneration at the neuromuscular junction in SMA and axon pruning at the neuromuscular junction during developmental synapse elimination [27], we wanted to establish whether motor neurons that are more susceptible to SMA display different dynamics during normal developmental synapse elimination.

**Figure 6:**
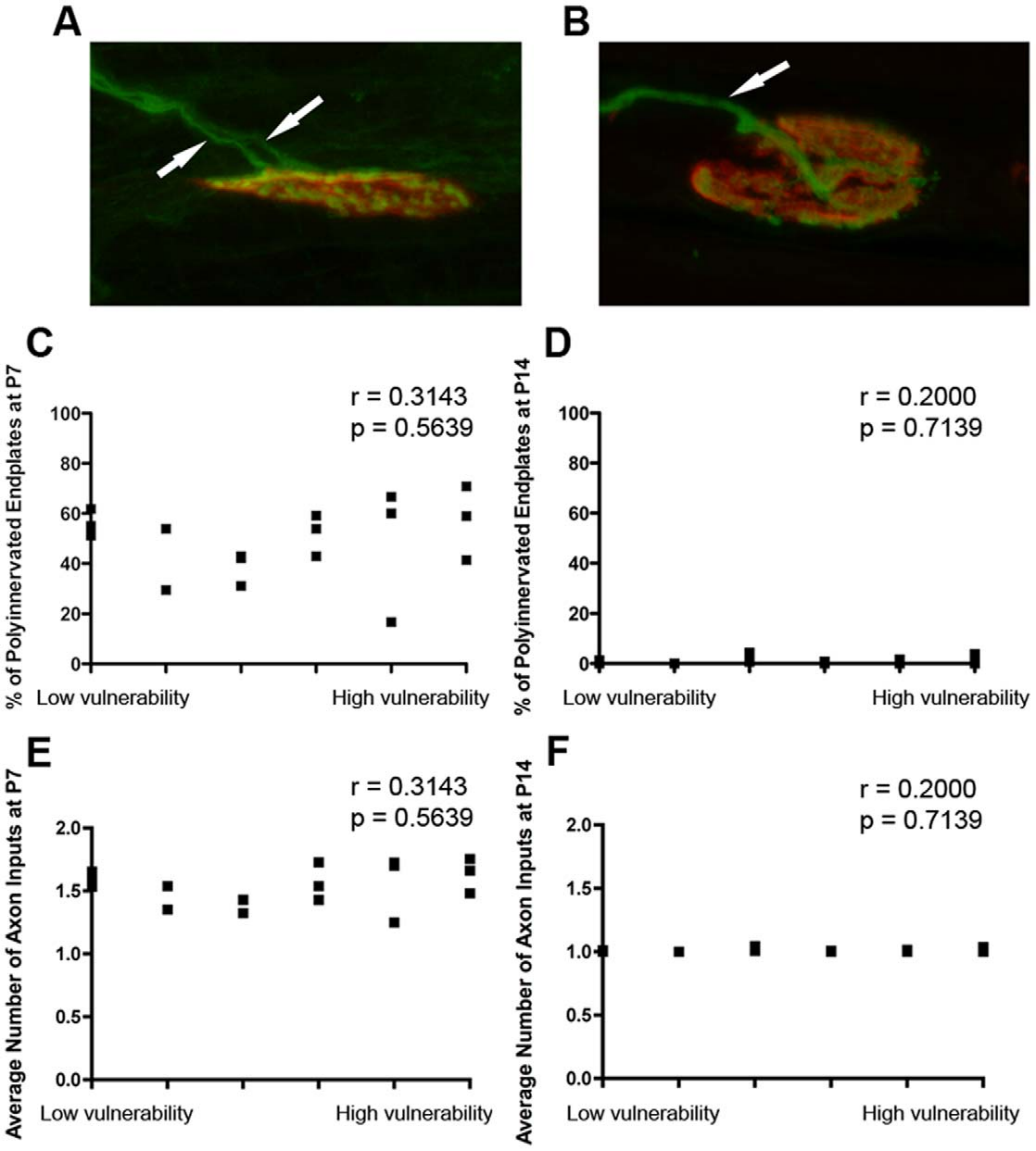
The relative susceptibility of a motor neuron to SMA is not determined by differences in intrinsic remodelling capabilities. A/B – Representative confocal micrographs showing immunohistochemically labelled neuromuscular junctions from a littermate control SMA mouse (*Smn+/−;SMN2*) at P7 (A) and P14 (B). White arrows in A show two axonal inputs (green) converging onto a single motor endplate (red), illustrating a polyneuronally innervated neuromuscular junction (the ‘immature’ state). The white arrow in B shows how a single axonal input is innervating the motor endplate, illustrating a mononeuronally innervated neuromuscular junction (the ‘mature’ state). C/D – Scatter plots showing the incidence of polyneuronally innervated endplates at P7 (C) and P14 (D) across a range of vulnerable and disease-resistant muscles in healthy littermate controls. There was no significant correlation between the percentage of polyinnervation (as a marker of the rate of synaptic remodelling during synapse elimination) and the relative susceptibility of the motor neuron at either P7 or P14 (P>0.05, Spearman correlation analysis; N = 3 mice per age group). E/F – Scatter plots showing the average number of axon inputs to motor endplates at P7 (E) and P14 (F) across a range of vulnerable and disease-resistant muscles in healthy littermate controls. As for the assessment based on percentages of polyinnervation, there was no significant correlation between the average number of axon inputs (as a marker of the rate of synaptic remodelling during synapse elimination) and the relative susceptibility of the motor neuron at either P7 or P14 (P>0.05, Spearman correlation analysis; N = 3 mice per age group). Note that, as expected, statistical analyses on C and D gave identical results to those on E and F as data were generated from the same experimental material.

Synapse elimination rates were determined across a range of vulnerable and disease-resistant muscle by quantifying the number of axonal inputs to individual motor endplates in healthy littermate control mice at P7 and P14 (Figure 6A–B). Quantitative analysis showed that there was no significant correlation between the percentage of polyinnervated neuromuscular junctions at P7 or P14 and the level of susceptibility to SMA (Figure 6C–D), or between the average number of axon inputs to neuromuscular junctions at P7 or P14 and the level of susceptibility to SMA (Figure 6E–F). Thus, motor neurons that were more susceptible to degeneration in SMA did not have different capabilities for intrinsic remodelling compared to those that were resistant to degeneration.

### Motor Neuron Vulnerability does not Correlate with the Number of Terminal Schwann Cells at the Neuromuscular Junction

Terminal Schwann cells (tSCs) are a specialised form of glial cell that overlies the nerve terminal of the motor neuron at the neuromuscular junction. It is well documented that tSCs are intricately involved in the development, maintenance, and stability of neuromuscular junctions [28]. We therefore sought to determine whether there was a difference in the complement of tSCs associated with neuromuscular junctions between motor neurons that are vulnerable and disease resistant in SMA.

**Figure 7:**
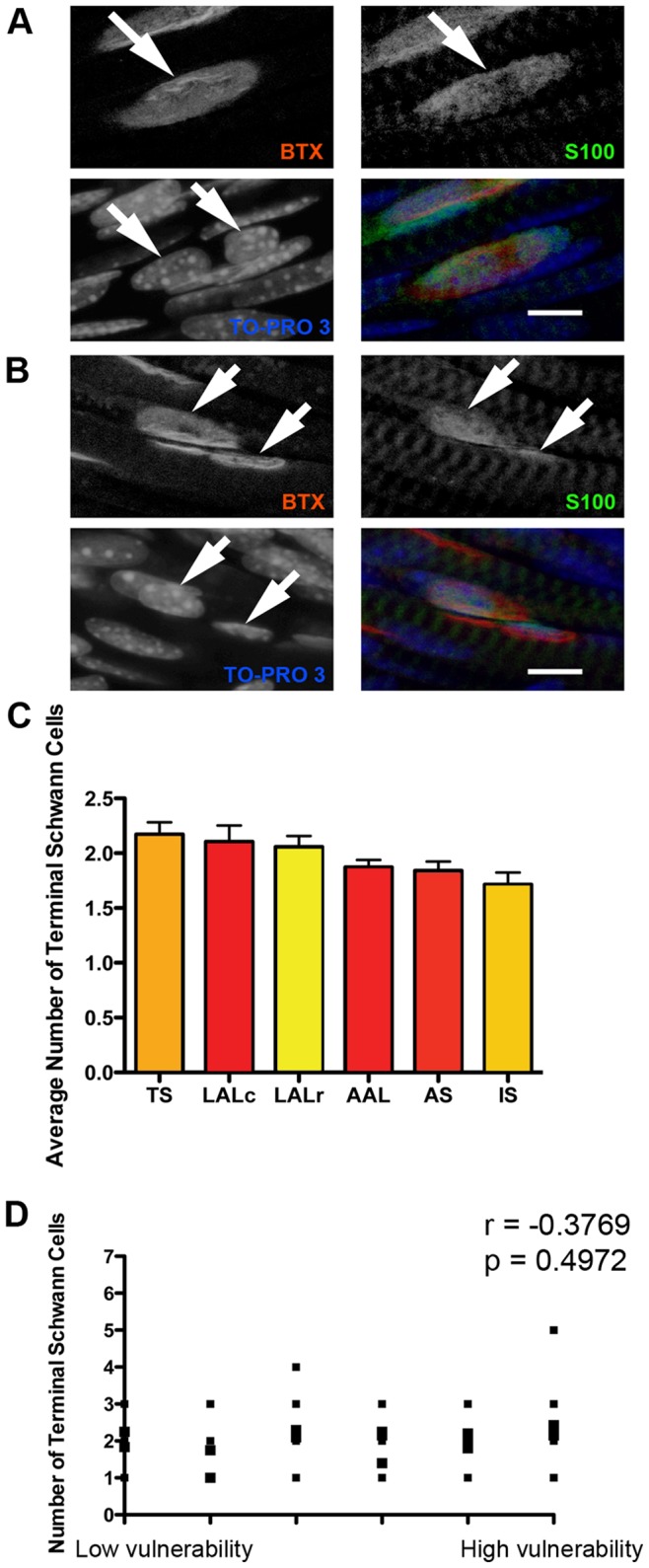
The number of terminal Schwann cells at the neuromuscular junction does not influence the relative susceptibility of motor neurons in SMA. A/B – Example confocal micrographs showing immunohistochemically labelled terminal Schwann cells (S100; green) at neuromuscular junctions in the LALr (A) and AAL (B) muscles. Nuclei were labelled with TOPRO-3 (blue) and motor endplates were labelled with bungarotoxin (red). The bottom left panel of A and B shows a merge of all three individual channels. Images were acquired on a confocal microscope using sequential capture to ensure no bleed-through from one channel to the next. The arrows in A show a single motor endplate (in the BTX channel), with clear terminal Schwann cell cytoplasm above it (in the S100 channel), but with two nuclei present within the S100 footprint (TO-PRO3 channel). This NMJ was therefore assessed to have 2 associated terminal Schwann cells. The arrows in B show two distinct motor endplates (in the BTX channel), each with clear terminal Schwann cell cytoplasm above it (in the S100 channel), but with only one nucleus present within the S100 footprint (TO-PRO3 channel) at each NMJ. These NMJs were therefore assessed to have 1 associated terminal Schwann cell each. Scale bars = 5 µm. C – Bar chart (mean±SEM) showing the mean number of terminal Schwann cells (tSCs) per NMJ in a range of muscles from P5 healthy littermate control mice (N = 3 mice). D – Scatter plot showing the number of tSCs per NMJ in each muscle examined, plotted against the relative vulnerability of motor neurons innervating that muscle. Statistical analysis showed that there was no significant correlation between the number of tSCs per NMJ and the susceptibility of the motor neuron in SMA (P>0.05, Spearman correlation analysis).

tSCs were immunohistochemically labelled in whole-mount P5 muscles from wild-type P5 mice using primary antibodies raised against S100 protein and the nuclear marker TO-PRO-3 (Figure 7A–B). There was no qualitative difference observed between tSCs in all muscles examined: all neuromuscular junctions had tSCs capping the synapse, with only a minimal extension of tSC cytoplasm beyond the boundaries of the motor endplate. Quantitative analyses of tSCs revealed no correlation between the mean number of tSCs at neuromuscular junctions and the relative susceptibility of the motor neuron to SMA (Figure 7C–D).

## Discussion

The current study demonstrates that, in sharp contrast to ALS, morphological characteristics of motor neurons do not determine their relative susceptibility to degeneration in SMA. Neither the position of the innervated muscle in the body, nor the muscle fibre type being innervated, modulated the susceptibility of a motor neuron. Similarly, we could find no correlation between factors such as motor unit size, total length of intramuscular axonal arbors, motor unit branching patterns, numbers of synaptic glia and intrinsic remodelling capabilities, in healthy young adult mice, and the relative susceptibility of some motor neurons in SMA.

The clear difference between our findings in a mouse model of SMA and previous studies of ALS patients and mouse models [19], [29], [30] was somewhat surprising. In particular, given that there is a growing body of evidence suggesting that the molecular and biochemical pathways underlying SMA and ALS are linked [13]–[15], it might have been expected that factors underlying motor neuron susceptibility would similarly be shared between the two conditions. The most parsimonious explanation for our findings is that motor neurons affected in SMA are targeted during the early stages of life, whereas motor neuron pathology in ALS is mostly associated with the later years of life. The relative contribution of morphological characteristics to the vulnerability of motor neurons in ALS may therefore represent factors that only manifest in the aging nervous system [20].

As morphological characteristics of motor neurons do not appear to be a major determinant of their relative susceptibility to degeneration in SMA, it remains unclear what factors are regulating their vulnerability. One possibility is that intrinsic differences persist between pools of vulnerable and disease-resistant motor neurons at a molecular level. The key determining factor of a motor neuron’s relative susceptibility to degeneration in SMA could therefore be dependent upon baseline levels of SMN protein. It has recently been established that the splicing efficiency of exon 7 in the *SMN2* gene is lower in motor neurons compared to other cells in the spinal cord [31], and perhaps further differences in splicing efficiency exists between distinct pools of motor neurons. Alternatively, it is possible that other molecular differences, such as intrinsic levels of neuroprotective factors, could account for the relative susceptibility of some motor neurons.

Although intrinsic differences in the molecular composition of vulnerable and disease-resistant motor neurons may contribute to their relative susceptibility to degeneration, our current findings also raise the possibility that interactions between motor neurons and other cell types may also be important. For example, intrinsic defects in skeletal muscle have been identified in SMA [32]. It is possible that intrinsic pathological changes in skeletal muscle pathology may have consequences for the stability of innervating motor neurons [33]. Similarly, deficiencies in central inputs onto motor neurons in the spinal cord have been identified in SMA mouse models [34], [35]. Differences in sensory-motor connectivity between vulnerable and disease-resistant motor neurons may therefore be an underlying cause of differential susceptibility. It is also possible that interactions between motor neurons and glial cells in the spinal cord and/or peripheral nerve influence their relative vulnerability. Studies of ALS have revealed potentially important roles for astrocytes, microglia and Schwann cells [36]–[37]. These cell types are therefore worthy of further investigation in SMA. However, data from the current study suggests that at least one type of glial cell, terminal Schwann cells present at the NMJ junction, appear not to play a major role in mediating motor neuron susceptibility.

Our study highlights the importance of animal models for examining factors regulating disease pathogenesis in SMA. The decision to examine motor unit morphology in healthy young adult mice expressing YFP (a requirement to obtain strongly-labelled motor neurons in the absence of pathological stimuli) does leave open the remote possibility that subtle intrinsic morphological properties of motor neurons specific to SMA mouse models may contribute to relative vulnerability in disease. However, the finding that motor neurons in pre-symptomatic SMA mice are grossly indistinguishable from those in wild-type littermates [11], suggests that this is unlikely to be the case. The extent to which mouse models of SMA accurately reproduce the human condition also remains unclear [38], [39]. The ability to reproduce the kind of experimental data presented in the current study in human patients is obviously limited, but recent developments in human stem cell technology suggest that it may soon be possible to recreate motor units from motor neuron disease patients in a dish [40], [41]. Such experimental models may be particularly beneficial in identifying factors that render some motor neurons more susceptible to degeneration than others in SMA.
